# Ti_3_C_2_T_x_ MXene-Based Hybrid Photocatalysts in Organic Dye Degradation: A Review

**DOI:** 10.3390/molecules30071463

**Published:** 2025-03-26

**Authors:** Tank R. Seling, Mackenzie Songsart-Power, Amit Kumar Shringi, Janak Paudyal, Fei Yan, Tej B. Limbu

**Affiliations:** 1 Department of Chemistry and Biochemistry, North Carolina Central University, Durham, NC 27707, USA; tseling@eagles.nccu.edu (T.R.S.); ashringi@nccu.edu (A.K.S.); 2 Department of Physical and Applied Sciences, University of Houston-Clear Lake, Houston, TX 77058, USA; songsartpowem7559@uhcl.edu; 3 Department of Chemistry and Physics, McNeese State University, Lake Charles, LA 70605, USA; jpaudyal@mcneese.edu

**Keywords:** Ti_3_C_2_T_x_ MXene, dye degradation, photocatalysis, charge separation

## Abstract

This review provides an overview of the fabrication methods for Ti_3_C_2_T_x_ MXene-based hybrid photocatalysts and evaluates their role in degrading organic dye pollutants. Ti_3_C_2_T_x_ MXene has emerged as a promising material for hybrid photocatalysts due to its high metallic conductivity, excellent hydrophilicity, strong molecular adsorption, and efficient charge transfer. These properties facilitate faster charge separation and minimize electron–hole recombination, leading to exceptional photodegradation performance, long-term stability, and significant attention in dye degradation applications. Ti_3_C_2_T_x_ MXene-based hybrid photocatalysts significantly improve dye degradation efficiency, as evidenced by higher percentage degradation and reduced degradation time compared to conventional semiconducting materials. This review also highlights computational techniques employed to assess and enhance the performance of Ti_3_C_2_T_x_ MXene-based hybrid photocatalysts for dye degradation. It identifies the challenges associated with Ti_3_C_2_T_x_ MXene-based hybrid photocatalyst research and proposes potential solutions, outlining future research directions to address these obstacles effectively.

## 1. Introduction

Rapid industrialization has escalated the production of toxic and carcinogenic wastewater [1]. The discharge of untreated wastewater from textile, pharmaceutical, dyeing, and paper printing industries significantly increases dye concentrations in water sources, posing a severe environmental threat [2]. These wastewaters contain chromophores and auxochromes, which color water, block sunlight, and disrupt photosynthesis, thereby harming aquatic ecosystems [3]. Discharged dyes are classified as cationic (e.g., methylene blue (MB), crystal violet (CV), rhodamine B (RhB)), which carry positive charges, are prevalent in textiles, and present toxicity risks, or anionic (e.g., acid orange 7 (AO7), acid blue 92 (AB92), Congo red (CR)), which carry negative charges, are water soluble, and are difficult to remove [4]. Various degradation methods, including membrane filtration, oxidation, adsorption, and photocatalysis, have been successfully developed and applied to address this issue [5,6].

Photocatalysis stands out as a highly effective degradation method due to its eco-friendliness and cost-effectiveness in treating organic pollutants [7]. Consequently, extensive research has focused on developing photocatalytic materials with wide energy bandgaps, abundant surface-active sites, and efficient charge separation capabilities to optimize dye photodegradation [8]. While single-component semiconducting photocatalysts like TiO_2_, ZnO, ZnS, SnO_2_, graphitic carbon nitride (g-C_3_N_4_), and CdS have been widely employed [9,10], their photocatalytic activity is often limited by poor visible light absorption, rapid charge recombination, and low electron–hole mobility [11,12]. To address these limitations and enhance photocatalytic performance, various strategies have been explored, including heterojunction construction [13], element doping [14,15], and co-catalyst introduction [14].

Modifying semiconducting materials with a co-catalyst is an effective strategy to enhance their photocatalytic activity [16]. A co-catalyst facilitates charge separation by preventing electron–hole recombination, a common limitation in photocatalysis. Materials such as graphene [17], carbon nanotubes [18], and carbon quantum dots [19] have been utilized as co-catalysts to improve dye photodegradation. While significant progress has been achieved using these co-catalysts, they each have certain limitations. Graphene and carbon nanotubes face challenges due to their scarcity of functional groups [20], while carbon quantum dots are hindered by their relatively low conductivity and scalability issues [21]. Challenges such as insufficient surface functional groups, which restrict chemical bonding between the photocatalyst and co-catalyst, and lower electrical conductivity, which impedes charge migration, can hinder photocatalytic efficiency. Consequently, there remains a need for efficient and cost-effective co-catalysts to optimize the photocatalytic degradation of organic dyes.

MXenes, a class of two-dimensional (2D) layered materials composed of transition metal carbides, nitrides, or carbonitrides with the general formula M_n+1_X_n_ (where M is a transition metal like Sc, Ti, Zr, Hf, V, Nb, Ta, or Mo, and X is carbon, nitrogen, or carbonitride), are promising for hybrid photocatalyst fabrication [22,23,24]. Their lower Fermi level compared to most studied semiconductors, abundant terminal functional groups, excellent metallic conductivity, and exposed terminal metal sites contribute to this potential. Specifically, the presence of abundant functional groups such as -OH, -O, and -F on Ti_3_C_2_T_x_ MXene facilitates strong interfacial chemical bonding with semiconductors, enabling the formation of a Schottky junction. This junction acts as an electron trap, effectively suppressing photoexcited electron–hole pair recombinations [25]. Furthermore, the excellent metallic conductivity of Ti_3_C_2_T_x_ MXene ensures rapid charge carrier migration, promoting efficient separation of photogenerated electrons and holes [26]. Lastly, the exposed terminal metal sites enhance reactivity compared to carbon-based materials, making Ti_3_C_2_T_x_ MXene a highly effective co-catalyst.

While Ti_3_C_2_T_x_ MXene exhibits limited standalone photocatalytic activity, requiring doping and UV radiation for optimal function [27], it plays a crucial role in composite photocatalysts. Beyond acting as a photogenerated charge acceptor co-catalyst, its large surface area and surface functionalities provide an excellent platform for the uniform growth, size control, and fine dispersion of photocatalysts, thereby exposing more surface-active sites [22,28,29,30]. Moreover, Ti_3_C_2_T_x_ MXene possesses distinctive physical and chemical properties, including high electrical conductivity, excellent hydrophilicity, mechanical stability, ion intercalation ability, and tunable surface functionalization [31,32,33]. These attributes render it an ideal component for composite photocatalyst materials. The integration of Ti_3_C_2_T_x_ MXene with semiconducting materials has yielded hybrid photocatalysts with micro/nano architectures and multi-junction nanocomposites. These hybrids leverage synergistic interactions between Ti_3_C_2_T_x_ MXene and conventional semiconductors or metal nanostructures, leading to enhanced charge separation and reduced recombination rates [23,34]. Consequently, Ti_3_C_2_T_x_ MXene-based hybrid photocatalysts demonstrate high effectiveness for the photodegradation of organic pollutants [35].

This review comprehensively explores Ti_3_C_2_T_x_ MXene-based hybrid photocatalysts for the degradation of cationic and anionic organic dyes. It details the synthesis of Ti_3_C_2_T_x_ MXene, its integration into hybrid photocatalysts, its role and effectiveness in dye degradation, and the underlying mechanisms. Furthermore, it discusses current challenges and emerging opportunities, providing insights into potential future research pathways.

## 2. Ti_3_C_2_T_x_ MXene and Synthesis Methods

### 2.1. Introduction to Ti_3_C_2_T_x_ MXenes

MXenes, first synthesized by Naguib et al. in 2011, are 2D crystals of transition metal carbides, nitrides, or carbonitrides [36]. These 2D materials are produced via top-down approaches, starting with their three-dimensional parent materials, MAX phases. MAX phases, represented by the general formula M_(*n*+1)_AX*_n_* (where *n* = 1, 2, or 3), consist of an early transition metal M (e.g., titanium, niobium, molybdenum, tantalum, vanadium, chromium), an A-group element A (primarily groups 13 and 14), and carbon, nitrogen, or carbonitride X layers [37]. Chemical etching removes the A layers from the MAX phase, resulting in the formation of 2D MXene crystals. Due to the predominantly aqueous synthesis methods, the M elements on the resulting MXene surfaces are terminated with functional groups, represented by Tx. These terminated MXenes are denoted by the general formula M_(*n*+1)_X*_n_*T*_x_* (where *n* = 1, 2, or 3), with T_x_ typically representing -OH, -O, or -F surface terminations [38,39,40,41,42].

MXenes have garnered significant attention due to their exceptional properties, including hydrophilicity, high electrical conductivity, and tunable bandgaps through surface termination modifications [42,43]. Ti_3_C_2_T_x_, the inaugural member of the MXene family, remains the most extensively researched due to its durability, the relatively simple process of etching aluminum layers from its MAX phase precursor, and its remarkable physical and chemical characteristics [36,44,45]. Consequently, leveraging their unique properties, numerous other MXene species have been explored for a wide range of applications.

### 2.2. Synthesis of Ti_3_C_2_T_x_ MXenes

Hydrofluoric acid (HF) etching was the pioneering technique for transforming MAX phases into MXenes [36]. Despite its simplicity and ability to yield high-quality MXenes [33], making it a widely used method, HF’s extreme toxicity and corrosiveness pose significant drawbacks [46]. To mitigate these risks, alternative methods, such as in situ HF generation via a reaction between LiF and HCl, have been developed. This approach also facilitates Li^+^ cation intercalation between MXene layers [47]. A characteristic outcome of HF etching is the formation of MXene layers with surface terminations, primarily -O, -OH, and -F, which enable surface engineering and bandgap tuning through modification of the termination type and quantity [48]. Another modified etching technique is the molten salt method, which involves reacting MAX phases with a Lewis acidic molten salt at elevated temperatures [39]. Similar to HF etching, an intercalant is utilized; however, the molten salt method allows for greater control over surface terminations during synthesis. For instance, -F terminations produced by HF etching can degrade the electrochemical performance of Ti_3_C_2_T_x_ electrodes in supercapacitors. To address this, Guo et al. [49] employed a one-step LiCl-KCl-K_2_CO_3_ molten salt etch and delamination process to replace -F terminations with -O, reducing the -F content from 11.23 to 3.43 atomic percent. This resulted in improved specific capacity, capacitance retention, conductivity, and electrochemical activity-specific surface area.

The hydrothermal etching method was developed as a safer and more environmentally friendly alternative to the highly corrosive and hazardous HF etching process. This technique enables efficient exfoliation and the production of high-quality MXene flakes, offering the advantages of larger interlayer spacing and improved delamination properties. Hydrothermal etching utilizes high-pressure and high-temperature conditions in an aqueous MXene solution to produce high-purity multilayer MXenes [46]. For example, Peng et al. [33] synthesized Ti_3_C_2_T_x_ MXenes using this technique by reacting MAX phases with HCl and HCl + NaBF_4_ solutions, followed by heating in an autoclave. The resulting MXenes were delaminated using dimethyl sulfoxide (DMSO) and sonication. XRD analysis revealed that hydrothermal etching was significantly more effective at removing aluminum compared to traditional HF etching. Furthermore, dye adsorption studies with cationic MB and anionic methyl orange (MO) demonstrated that hydrothermally etched Ti_3_C_2_T_x_ exhibited superior adsorption performance, with lower residual dye concentrations compared to non-hydrothermal counterparts. Some hydrothermal methods incorporate microwaves to excite reagents and reduce reaction temperature and time [50].

The resulting flakes were cleaned, delaminated using tetramethylammonium hydroxide, cleaned again, and then incorporated into a composite with reduced graphene oxide, which effectively degraded dyes via photolysis [51]. Cao et al. [50] utilized microwave hydrothermal etching to rapidly create and oxidize Ti_3_C_2_T_x_ MXene nanosheets. After cleaning, this Ti_3_C_2_T_x_ was combined with TiO_2_ and CdZnS, again using microwave hydrothermal methods, to synthesize a synergistic photocatalytic semiconductive heterojunction. This heterojunction degraded RhB dye molecules by 29.33% within 90 min, a 31.17-fold improvement compared to single Ti_3_C_2_ [52]. To drastically reduce etching time (from approximately 2–3 days to 45 min) and eliminate -F terminations, Latif et al. [53] applied 5 to 30 M concentrations of NaOH etchant to Ti_3_AlC_2_ in a microwave hydrothermal reaction. Higher NaOH concentrations resulted in synthesized Ti_3_C_2_T_x_ MXene, with a 0.46% aluminum content, as determined by XRD, and a semiconductive bandgap energy of 1.30 to 1.60 eV.

## 3. Design and Fabrication of Ti_3_C_2_T_x_ MXene-Based Hybrid Photocatalysts

A wide range of methods have been employed to fabricate Ti_3_C_2_T_x_ MXene-based hybrid photocatalysts, involving the integration of Ti_3_C_2_T_x_ MXene nanosheets with diverse nanomaterials. As a result of their chemical synthesis, Ti_3_C_2_T_x_ MXene materials naturally acquire surface terminations, and their tunable surface chemistry provides potential pathways for hybridization with various material classes through covalent and non-covalent interactions [54]. The redox behavior of titanium (Ti) has been leveraged to develop TiO_2_-based semiconductors on the MXene surface, where MXene serves as a growth platform. The presence of hydrophilic surface functional groups such as -O-, -OH, and -F is theoretically expected to enhance chemical bonding with other semiconductor photocatalysts. However, the development of hybrid photocatalysts utilizing the MXene functional terminations through covalent bonding requires state-of-the-art approaches, and hence, most studies have primarily utilized non-covalent approaches, where charge interactions or hydrophilic groups act as adsorption or anchoring sites for metal salts, semiconductors, and other materials [55,56].

**Figure 1:**
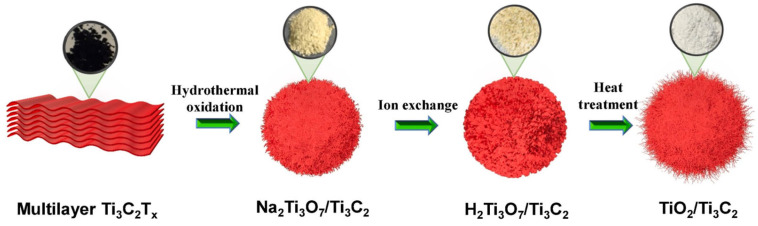
Schematic illustration of the TiO_2_/Ti_3_C_2_ hybrid synthesis process through partial oxidation of Ti_3_C_2_. Reproduced with permission from [57].

Tran et al. [57] fabricated TiO_2_/Ti_3_C_2_T_x_ composites through an in situ partial oxidation of Ti_3_C_2_T_x_, resulting in microscale safflower-like structures composed of TiO_2_/Ti_3_C_2_T_x_ heterostructure nanorods. This transformation involved sequential hydrothermal oxidation, alkalization, ion exchange, and heat treatment, during which layered MXene flakes were fragmented into nanoparticles, from which TiO_2_/Ti_3_C_2_T_x_ nanorods grew radially. A schematic of this synthesis process is shown in Figure 1.

The resulting TiO_2_/Ti_3_C_2_T_x_ heterostructures demonstrated exceptional photocatalytic properties. Their photocurrent was ten times greater than that of pristine MXene. Furthermore, the photocatalytic degradation efficiency of rhodamine B (RhB) reached 95%, a significant improvement over the 19% achieved by MXene alone. Even after four cycles, the degradation efficiency remained above 95%, indicating excellent stability. This superior performance was attributed to the rapid generation of TiO_2_ carriers, suppressed charge carrier recombination, and enhanced light absorption due to the porous safflower-like structure. Similarly, Quyen et al. [58] employed a novel synthesis approach to create TiO_2_@Ti_3_C_2_T_x_ nanoflowers with a porous 3D framework derived from 2D Ti_3_C_2_T_x_ MXenes via hydrothermal oxidation combined with calcination. This in situ transformation converted the initially conductive Ti_3_C_2_T_x_ MXene into a semiconductor, forming a TiO_2_@Ti_3_C_2_T_x_ heterojunction. Compared to the 36% degradation rate of pure TiO_2_ for RhB, the TiO_2_@Ti_3_C_2_T_x_ composite achieved an impressive 97% degradation within 40 min of light irradiation. This enhancement was attributed to the electronic structure of Ti_3_C_2_T_x_, whose Fermi level is lower than that of TiO_2_. Upon light excitation, photoinduced electrons transferred from the valance (CB) of TiO_2_ to metallic Ti_3_C_2_T_x_, leaving holes in the valence band (VB) of TiO_2_. The resulting Schottky barrier at the interface suppressed electron diffusion back into TiO_2_, thereby reducing electron–hole recombination and improving photocatalytic efficiency. In a similar vein, an in situ solvothermal process was utilized to prepare facet-exposed TiO_2_/Ti_3_C_2_T_x_ [59]. Since the exposed crystal planes of TiO_2_ can effectively capture photogenerated holes and facilitate rapid migration to the Ti_3_C_2_T_x_ surface, the photocatalytic performance towards methyl orange was significantly superior compared to TiO_2_ and Ti_3_C_2_T_x_ alone.

**Figure 2:**
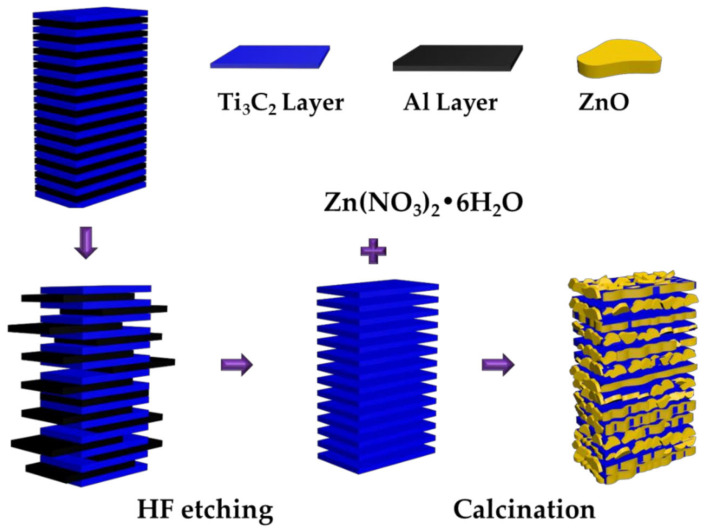
Schematic illustration of the ZnO/Ti_3_C_2_T_x_ hybrid photocatalyst synthesis process. Reproduced with permission from [60].

Calcination, a simple method for preparing Ti_3_C_2_T_x_ MXene composites, involves heating powders of two materials at elevated temperatures. As illustrated in Figure 2, varying amounts of Ti_3_C_2_T_x_ MXene powders were thoroughly dispersed in a Zn^2+^-containing solution [60]. The resulting mixture was heated in an oven to evaporate the solvent. Subsequently, the powder was calcined at 550 °C for 4 h, with a heating rate of 5 °C/min in ambient air to synthesize ZnO/Ti_3_C_2_T_x_ hybrid structures. Compared to pristine ZnO, the hybrid structure exhibited reduced photoluminescence intensity, an enhanced Brunauer–Emmett–Teller (BET) surface area, and improved photocatalytic degradation efficiency for MO and RhB.

**Figure 3:**
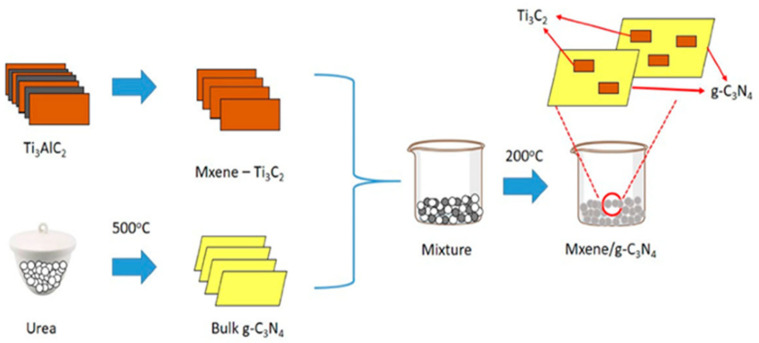
Schematic illustration of the Ti_3_C_2_T_x_ MXene/g-C_3_N_4_ photocatalyst synthesis process using the wet impregnation method. Reproduced with permission from [6].

The wet impregnation method, where one material is deposited onto a solid support, can be used to prepare Ti_3_C_2_T_x_ MXene composites. Nasri et al. [6] mixed g-C_3_N_4_ and Ti_3_C_2_T_x_ MXene powders in varying weight proportions and sonicated the mixture until a slurry formed. The resulting slurry was dried overnight at 60 °C to obtain the Ti_3_C_2_T_x_ MXene/g-C_3_N_4_ composite photocatalyst. Figure 3 illustrates the fabrication process of this composite. They found that the 1 wt% Ti_3_C_2_T_x_ MXene/g-C_3_N_4_ heterostructure exhibited higher photocatalytic activity for methylene blue degradation compared to pure g-C_3_N_4_. This enhanced activity was attributed to intimate interfacial contact (observed via field emission scanning electron microscopy (FESEM) analysis), efficient photo-charge carrier transfer, and a larger BET surface area.

Electrostatically driven self-assembly is a facile approach for synthesizing MXene composites, particularly in solutions. Cai et al. [61] synthesized an Ag_3_PO_4_/Ti_3_C_2_T_x_ MXene Schottky catalyst that exhibited prominent photodegradation performance towards various organic dyes, including methyl orange and 2,4-dinitrophenol. In their process, Ti_3_C_2_T_x_ sheets were first dispersed in deionized (DI) water using sonication, followed by the addition of an AgNO_3_ aqueous solution to the Ti_3_C_2_T_x_ suspension under vigorous stirring. Subsequently, a Na_2_HPO_4_ aqueous solution was added dropwise to the mixture and stirred for 2 h. The resulting precipitate was washed multiple times with DI water and dried in a vacuum oven at 80 °C overnight. The negatively charged surface of Ti_3_C_2_T_x_, due to abundant surface termination groups, facilitated its interaction with Ag_3_PO_4_. The apparent rate constant for 2,4-dinitrophenol degradation with Ag_3_PO_4_/Ti_3_C_2_T_x_ was 2.5 times higher than that of Ag_3_PO_4_/RGO and 10 times higher than that of Ag_3_PO_4_ alone. This enhanced photocatalytic activity of Ag_3_PO_4_/Ti_3_C_2_T_x_ was attributed to the sufficient and close interfacial contact between Ag_3_PO_4_ and Ti_3_C_2_T_x_, unidirectional electron flow trapped by Ti_3_C_2_T_x_ across the Schottky barrier, and the stronger redox reactivity of surface metal Ti sites on Ti_3_C_2_T_x_.

**Figure 4:**
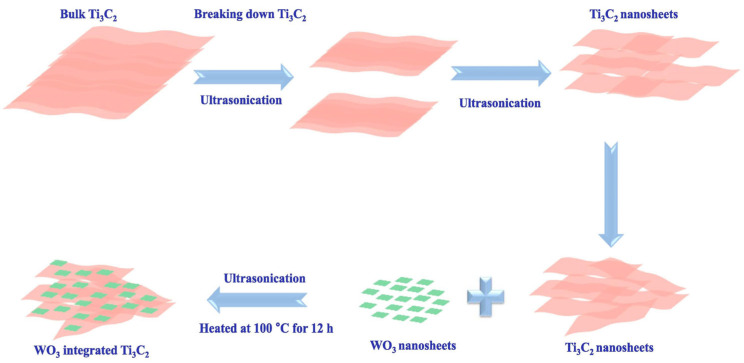
Schematic illustration of the 2D/2D WO_3_/Ti_3_C_2_T_x_ heterojunction formation process. Reproduced with permission from [62].

Ultrasonic forces have been employed in the fabrication of Ti_3_C_2_T_x_ MXene-based composites to disrupt electrostatic attractions and van der Waals interactions. For example, Lee et al. [62] prepared 2D/2D WO_3_/Ti_3_C_2_T_x_ heterojunction composites (Figure 4). WO_3_ nanosheets were dispersed in deionized (DI) water, followed by the addition of varying amounts of Ti_3_C_2_T_x_ nanosheets. The resulting suspension was sonicated, where cavitation bubbles generated localized high-temperature and high-pressure spots, facilitating physical and chemical interactions between WO_3_ and Ti_3_C_2_T_x_. This process enabled the effective integration of WO_3_ nanosheets with Ti_3_C_2_T_x_ structures. The final suspension was dried at 100 °C for 12 h in an electric oven. The WO_3_/Ti_3_C_2_T_x_ heterojunction demonstrated significantly higher photoexcited carrier transfer and separation efficiency, resulting in exceptional photocatalytic performance for methylene blue (MB) degradation under visible light. Furthermore, photoelectrochemical analysis of WO_3_/Ti_3_C_2_T_x_ revealed improved charge carrier mobility, effectively reducing the carrier transport barrier between WO_3_ and Ti_3_C_2_T_x_. A similar approach has been used to synthesize Ti_3_C_2_T_x_/CuFe_2_O_4_ nanohybrids [63].

A similar process has been used to prepare ZnS nanoparticle/layered MXene sheets [64]. Likewise, manganese oxide-decorated 2D Ti_3_C_2_T_x_ MXene containing MnO_2_ nanopetals were also synthesized using the ultrasonic approach [65]. The synergistic effect of these two nanomaterials inhibited electron–hole pair recombination and improved surface activity. The nanocomposite exhibited high photocatalytic ability, degrading approximately 99% of MB within 30 min.

The sol-gel method employs metal alkoxide precursors to form gels via hydrolysis, typically at lower temperatures, followed by calcination. For instance, Iqbal et al. [66] utilized a double-solvent solvothermal method to synthesize BiFeO_3_ (BFO)/Ti_3_C_2_T_x_ nanohybrid. They separately ultrasonicated Ti_3_C_2_T_x_ MXene in deionized (DI) water and BFO nanoparticles in a mixture of acetic acid and ethylene glycol. The resulting solutions were combined and transferred to a Teflon-lined steel autoclave for solvothermal synthesis, where the mixture was heated at 160 °C for 2 h. The final product was washed and dried at 80 °C for 3 h. This nanohybrid exhibited a high BET surface area of 147 m^2^ g^−^^1^, a low band gap of 1.96 eV, and a reduced recombination time. These attributes contributed to the material’s superior photocatalytic performance, demonstrated by the degradation of CR dye within a 42 min period under visible light irradiation. A similar process was used to prepare La- and Mn-co-doped BFO nanoparticles embedded in Ti_3_C_2_T_x_ sheets [67].

**Figure 5:**
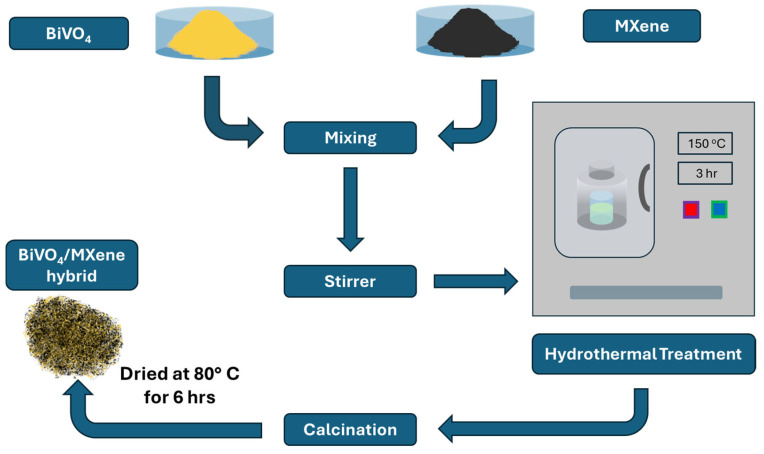
Flow chart of the synthesis process for BiVO_4_/Ti_3_C_2_T_x_ nanocomposite [70].

The solvothermal process, which involves reactions in a solvent under elevated temperatures and pressures within a sealed system, has been employed to synthesize MXene nanocomposites. For instance, Zheng et al. [68] synthesized SnO_2_/Ti_3_C_2_T_x_ composites, and Zhou et al. [69] prepared CeO_2_/Ti_3_C_2_T_x_ nanocomposites using CeO_2_ nanorods on Ti_3_C_2_T_x_ sheets. These nanocomposites demonstrated enhanced photocatalytic activity for RhB photodegradation under UV-light irradiation compared to pure CeO_2_ semiconductors and Ti_3_C_2_T_x_. This enhancement was attributed to the composite’s narrower bandgap compared to CeO_2_, facilitating improved solar energy utilization and resulting in high pollutant degradation efficiency [69]. A similar process was utilized to prepare BiVO_4_/Ti_3_C_2_T_x_ nanocomposites [70]. This nanohybrid was synthesized by first preparing a 0.005 g/mL Ti_3_C_2_T_x_ MXene solution in D.I. water via 15 min ultrasonication. Separately, 0.40 g of BiVO_4_ was dispersed in a 1:1 ethanediol–ethanoic acid mixture and ultrasonicated at 80 °C for 30 min. The solutions were then mixed, stirred for 1 h, and subjected to hydrothermal treatment at 150 °C for 3 h in a Teflon-lined autoclave. The product was washed with D.I. water and ethanol, then dried at 80 °C for 6 h, yielding a nanocomposite powder. The fabrication of BiVO_4_/Ti_3_C_2_T_x_ is illustrated in Figure 5.

**Figure 6:**
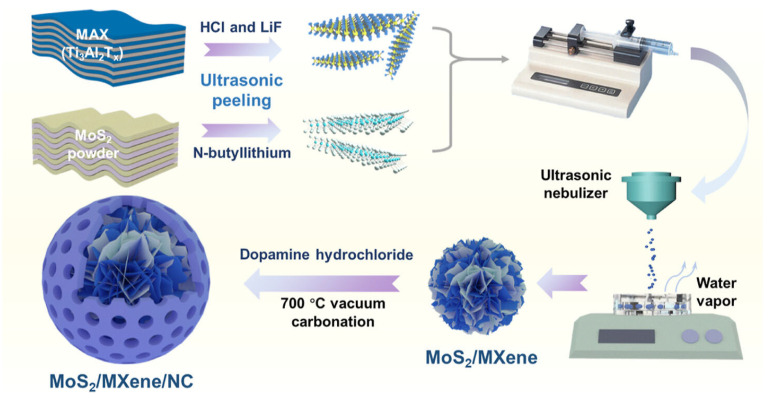
Schematic of the synthesis and fabrication of MoS_2_/Ti_3_C_2_T_x_ MXene/N-doped carbon composite microspheres. Reproduced with permission from [71].

Fan et al. [71] synthesized monolayer Ti_3_C_2_T_x_ MXene nanosheets through LiF-HCl etching, followed by washing, centrifugation, and ultrasonic exfoliation. MoS_2_ nanosheets were produced via lithium-ion etching with n-butyllithium under argon for 48 h, then neutralized, sonicated, and centrifuged. To fabricate MoS_2_/MXene/NC composite microspheres, MoS_2_ and MXene dispersions were ultrasonicated, nebulized into droplets, and self-assembled in liquid wax at 150 °C. Subsequently, the microspheres were coated with polydopamine (PDA) using dopamine hydrochloride in Tris buffer. Vacuum carbonization at 700 °C yielded composite microspheres with varying PDA coating times (4, 8, 12 h) and MoS_2_-to-MXene ratios (1:3, 1:5, 1:7), as detailed in Figure 6.

It was found that the Schottky junction and heterojunction between Ti_3_C_2_T_x_ and MoS_2_ significantly prolonged the recombination time of electron–hole pairs and broadened the visible light absorption range. Consequently, the nanohybrid exhibited enhanced photocatalytic activity towards MO. It was also found that TiO_2_/Ti_3_C_2_T_x_ MXene was synthesized via a hydrothermal reaction involving nano-TiO_2_ and Ti_3_C_2_T_x_ MXene nanosheets [72]. The improved photocatalytic activity was attributed to the suppression of electron–hole recombination, which facilitated electron accumulation and enhanced electron transfer from TiO_2_ to MXene. Hydrothermal treatment of Ti_3_C_2_T_x_ nanosheets and AgNO_3_ at 160 °C for 12 h, with a 2 °C/min heating rate, resulted in the fabrication of AgNPs/TiO_2_/Ti_3_C_2_T_x_ composite [73]. During this process, silver nanoparticles (AgNPs) were deposited on the MXene surface via the self-reduction of silver salts, where MXene acted as a redox agent, facilitating the nucleation and growth of spherical Ag nanoparticles on the MXene nanosheets. The photocatalytic performance of the oxidized form was significantly improved compared to the pristine form. AgNPs/TiO_2_/Ti_3_C_2_T_x_ also showed superior degradation efficiency for MB and RhB compared to pristine MXene. Bi_2_WO_6_/Ti_3_C_2_T_x_ was synthesized using a similar process involving heating layered Ti_3_C_2_T_x_ MXene and Bi(NO_3_)_3_·5H_2_O at 160 °C for 16 h.

## 4. Photocatalytic Degradation of Dyes Using Ti_3_C_2_T_x_ MXene Hybrids

Photocatalysis presents a relatively safe and cost-effective strategy for degrading hazardous organic pollutants [41,74,75]. Ti_3_C_2_T_x_ MXene, with its unique lamellar structure, remarkably high metallic conductivity, and excellent hydrophilicity, has emerged as a promising member of the MXene family. Its distinctive properties have enabled its application as a photocatalyst for environmental remediation [76,77] and as a co-catalyst for enhancing the photocatalytic degradation potential of composite photocatalysts [34,78]. Ti_3_C_2_T_x_ MXene-based hybrid photocatalysts offer several advantages, including improved charge separation, efficient atomic utilization, tunable bandgap, optimized morphology, enhanced electron transfer efficiency, and increased photocatalytic activity [79,80,81]. This review assesses the effectiveness of Ti_3_C_2_T_x_ MXene-based hybrid photocatalysts by comparing their performance to non-hybrid photocatalysts in the degradation of both cationic and anionic dyes (Table 1).

Table 1 summarizes the results of studies on the photocatalytic degradation of anionic dyes (MO, CR) and cationic dyes (MB, RhB). Dey and Ratan Das [81] achieved 95% degradation of MO within 300 min using CdS as a non-hybrid photocatalyst. In another study, Mohammad Jafri et al. [85] utilized TiO_2_ nanofibers, achieving 95.2% degradation of MB within 240 min, and Mary et al. [88] reported 97.6% degradation of CR in 75 min using ZnO. Similarly, Fang et al. [90] prepared g-C_3_N_4_, achieving 75% degradation of RhB in 180 min. The ZnO standalone photocatalyst required 1260 min (21 h) to degrade 40.88% of MB [84], while MoSe_2_ achieved only 8.44% degradation in 120 min (2 h) [89]. These prolonged durations and low efficiencies render such photocatalytic processes economically and temporally inefficient for the practical degradation of organic dye pollutants. Furthermore, pristine MXene photocatalysts exhibit lower dye degradation efficiencies. For example, Qu et al. [92] reported that the alkalized Ti_3_C_2_T_x_ demonstrated reduced photocatalytic performance compared to Ti_3_C_2_T_x_ MXene-based hybrid photocatalysts. Specifically, the alkalized Ti_3_C_2_T_x_ achieved degradation rates of only 17.3% for RhB and 2.8% for MO within 120 min.

Conversely, Ti_3_C_2_T_x_ MXene-based hybrid photocatalysts have demonstrated remarkable potential for degrading both cationic and anionic dyes under light irradiation. For example, a Ti_3_C_2_T_x_ MXene-based hybrid photocatalyst achieved a degradation efficiency of 99.7% for MO and 100% for RhB, as detailed in Table 2. These photocatalysts effectively decompose dye molecules into less harmful degradation products, showcasing their efficiency in addressing organic dye pollutants. This high efficiency can be attributed to the rich surface chemistry, tunable bandgap structures, high electrical conductivity, hydrophilicity, thermal stability, and large specific surface area with abundant active sites. These properties facilitate efficient dye adsorption and subsequent photocatalytic degradation. For instance, Nasri et al. [6] reported 100% degradation efficiency for MB using a Ti_3_C_2_T_x_/g-C_3_N_4_ hybrid within 180 min. This exceptional performance was attributed to the effective charge separation and transfer capabilities of Ti_3_C_2_T_x_ MXene, which significantly enhanced the generation of reactive species essential for dye degradation.

Ta et al. [60] demonstrated that ZnO/Ti_3_C_2_T_x_ achieved a 99.7% degradation efficiency for MO, while Bi_2_WO_6_/Ti_3_C_2_T_x_ effectively degraded RhB, with an impressive 99.9% efficiency within 20 min., Zhao and Cai [93] reported comparable results (see Table 2). These studies highlight the pivotal role of MXene-incorporated heterostructures, characterized by their high conductivity and ability to facilitate rapid electron transfer, which is essential for efficient photocatalysis [94]. Yao and Wang [95] demonstrated that MB achieved a 93.3% degradation effi-ciency within 160 min using a standalone TiO_2_ catalyst at a catalyst-to-dye ratio of 1.5:1. However, the MXene-based hybrid AgNPs/TiO_2_/Ti_3_C_2_T_x_ achieved 99% degrada-tion in just 30 min at a 2.5:1 catalyst-to-dye ratio, demonstrating superior efficiency in both time and degradation rate compared to non-hybrid photocatalysts [73].

Ti_3_C_2_T_x_ MXene-based hybrids represent a versatile and highly effective class of photocatalysts for the degradation of both anionic and cationic dyes, making them suitable for various environmental remediation applications. For example, Ti_3_C_2_T_x_ MXene-based nanocomposites prepared with Mn_2_O_3_ were evaluated for photocatalytic dye degradation under light, demonstrating efficient photocatalysis. The one-dimensional (1D) Mn_2_O_3_-Ti_3_C_2_T_x_ (20 wt%) nanocomposite achieved 100% degradation of MB within 25 min, effectively removing the dye [96]. Similarly, NiM-nO3/NiMn_2_O_4_-Ti_3_C_2_T_x_ MXene nanocomposites achieved 100% degradation of MB in 50 min, showcasing excellent dye removal efficiency [97]. Wang et al. [98] synthesized a Ti_3_C_2_T_x_/Bi_4_Ti_3_O_12_ heterojunction via a facile in-situ solvothermal method, demonstrating exceptional visible-light-driven photocatalytic performance by achieving 100% degradation of MO and RhB within 60 and 50 min, respectively (see Table 2). These results highlight the potential of Ti_3_C_2_T_x_ MXene-based hierarchical composites for water remediation, offering a sustainable approach for the degradation of anionic and cationic organic pollutants. In an independent study, Iqbal et al. [66] reported that the BiFeO_3_ (BFO)/Ti_3_C_2_T_x_ MXene hybrid achieved 100% degradation of CR in 42 min, highlighting its potential for photocatalysis applications.

It was observed that factors such as the synthesis method, pH, catalyst-to-dye ratio, concentration, and structure significantly influence the functionality and effectiveness of photocatalysts. Based on this evaluation, it is concluded that Ti_3_C_2_T_x_ MXene-based hybrid photocatalysts exhibit superior photocatalytic performance and efficiency in degrading organic pollutant dyes compared to non-hybrid photocatalysts.

**Table 2:** Ti_3_C_2_T_x_ MXene-based hybrid photocatalysts with photocatalytic degradation performance towards cationic and anionic dyes.

MXene-Based Hybrid Photocatalysts	Dyes	Type of Dyes (Based on Charge)	Degradation Percentage/(Time)	References
TiO_2_/Ti_3_C_2_T_x_	MO	Anionic	92 (50 min)	[59]
MoS_2_@Ti_3_C_2_	MO	Anionic	98 (60 min)	[99]
Ti_3_C_2_/TiO_2_/CuO	MO	Anionic	99 (80 min)	[100]
ZnO/Ti_3_C_2_T_x_	MO	Anionic	99.7 (50 min)	[60]
Ti_3_C_2_T_x_/Bi_4_Ti_3_O_12_	MO	Anionic	100 (60 min)	[98]
TiO_2_/Ti_3_C_2_ Mxene	MB	Cationic	96.44 (60 min)	[101]
AgNP_s_/TiO_2_/Ti_3_C_2_T_x_	MB	Cationic	99 (30 min)	[73]
Ti_3_C_2_/g-C_3_N_4_	MB	Cationic	100 (180 min)	[6]
NiMnO_3_/NiMn_2_O_4_-Ti_3_C_2_T_x_ MXene	MB	Cationic	100 (50 min)	[97]
1D Mn_2_O_3_-Ti_3_C_2_T_x_	MB	Cationic	100 (25 min)	[96]
Mn-codoped bismuth ferrite/Ti_3_C_2_	CR	Anionic	93 (30 min)	[67]
CoFe_2_O_4_@MXene	CR	Anionic	98.9 (30 min)	[102]
BiVO_4_/Ti_3_C_2_	CR	Anionic	99.5 (60 min)	[70]
BGFO-20Sn/MXene	CR	Anionic	100 (120 min)	[103]
BiFeO_3_ (BFO)/Ti_3_C_2_	CR	Anionic	100 (42min)	[66]
TiO_2_@Ti_3_C_2_	RhB	Cationic	97 (40 min)	[58]
BiOBr/TiO_2_/ Ti_3_C_2_T_x_	RhB	Cationic	99.8 (30 min)	[104]
Bi_2_WO_6_/Ti_3_C_2_	RhB	Cationic	99.9 (20 min)	[93]
ZnS/MXene	RhB	Cationic	100 (100 min)	[64]
Ti_3_C_2_T_x_/Bi_4_Ti_3_O_12_	RhB	Cationic	100 (50 min)	[98]

## 5. Computational Studies and Simulations

The investigation of Ti_3_C_2_T_x_ MXene-based hybrids for photocatalytic applications is significantly enhanced by computational tools and techniques, which complement experimental approaches. These methods offer insights into atomic-level phenomena, guide material design, and predict performance under varying conditions.

Chen et al. [101] investigated the electronic and optical properties of -F terminated, -O terminated, and termination-free Ti_3_C_2_ in an MXene nanosheet/TiO_2_ composite using density functional theory (DFT). Their study revealed that surface terminations reduced the density of electronic states, lowered conductivity, and enhanced stability compared to the termination-free MXene. DFT analysis also demonstrated the feasibility of electron transfer from TiO_2_ to Ti_3_C_2_ and identified the Schottky barrier at the interface between the two materials. Furthermore, computational modeling highlighted the synergy between the composite components, showing an extended range of light absorption, suppressed electron–hole recombination, and improved hole oxidation efficiency in the VB of TiO_2_. These factors significantly enhanced the photocatalytic performance of the Ti_3_C_2_/TiO_2_ composite, making it a promising candidate for photocatalytic applications, such as treating organic pollutants like dye molecules [101]. Lemos et al. utilized a computational model to evaluate the performance of a Ti_3_C_2_T_x_/TiO_2_ nanocomposite hybrid for photocatalyzed dye-sensitized solar cells. Through DFT calculations, they discovered that the anatase potential is reduced at the nanocomposite interface and that the nanocomposite exhibits improved photocarrier separation at the interface between the nanocomposite and the dye [105]. Furthermore, Yang et al. [106] conducted a computational analysis to confirm that a transition metal dichalcogenide/MXene photocatalyst hybrid, MoS_2_/Ti_3_C_2_, functions as a Schottky barrier. A Bader charge analysis revealed that the difference in work functions between MoS_2_ and Ti_3_C_2_, combined with a built-in electric field, facilitates the transfer of photogenerated electrons from MoS_2_ to the Ti_3_C_2_ electron sink. This efficient electron transfer enhances photocarrier separation, resulting in longer lasting photogenerated holes and exceptional photodegradation performance against rhodamine B dye in wastewater [106].

Several computational techniques have been employed to assess and optimize photocatalytic mechanisms in MXene-based composites for dye degradation applications. Liu et al. [107] developed a g-C_3_N_4_/Ti_3_C_2_ (CNTC) heterojunction by hybridizing 2D Ti_3_C_2_ MXene with three-dimensional (3D) g-C_3_N_4_ for enhanced photodegradation of RhB dye. This photocatalyst exhibited a high specific surface area (85.08 m^2^/g) and remarkable charge migration capabilities. To evaluate its improved photocatalytic performance, the researchers utilized DFT analysis to examine the differential charge density, electron distribution, and charge transfer dynamics between g-C_3_N_4_ and the Ti_3_C_2_ sink. The study revealed that the excellent conductivity of Ti_3_C_2_ stemmed from the overlap between the Fermi level and CB in the heterojunction. This led to the understanding that these 2D/3D heterojunctions significantly promote charge transfer and separation, which are essential for efficient photocatalysis. Additionally, the combination of a high specific surface area and abundant active sites makes the CNTC particularly effective for dye photodegradation [107]. Cheng et al. [108] synthesized a self-cleaning BiOCl-polypyrrole Ti_3_C_2_T_x_ MXene composite membrane with excellent photocatalytic activity and high flux, designed for filtering and degrading pollutants. The membrane’s performance was tested against various dyes. To gain deeper insight into the photocatalytic mechanisms, particularly charge separation and degradation, the researchers conducted DFT calculations. Density of state (DOS) simulations revealed that the chemisorption of oxygen into vacancies on the BiOCl surface generated superoxide radicals. These radicals enhanced the composite’s photocatalytic efficiency through redox interactions with dye molecules and other pollutants [108]. Wang et al. [109] synthesized an S-scheme Pt-MnO_2_/TiO_2_@Ti_3_C_2_T_x_ composite using an electrostatically self-assembled Ti-O-Mn bond and evaluated its oxidative photodegradation performance against MB, MO, and RhB dyes. To investigate the photocatalytic mechanisms, DFT calculations were performed, revealing that the Ti-O-Mn bond induced the formation of metastable Ti atoms and electrostatically adsorbed Mn^2+^ ions. This bond facilitated the separation of photoinduced carriers and optimized their transport pathways. Additionally, the DFT analysis identified the formation of S-scheme heterojunctions between MnO_2_ and TiO_2_ through the Ti-O-Mn bond, driving the flow of photogenerated carriers. These factors collectively enhanced the composite’s photocatalytic efficiency [109].

DFT has been employed in conjunction with the finite element method to explore ways to enhance the photocatalytic activity and membrane permeability of a novel MXene-based composite membrane, N-doped Bi_2_O_2_CO_3_@Ti_3_C_2_T_x_/Polyethersulfone, designed for oil/water separation and dye degradation [110]. Through DFT analysis of the electron distribution and band structure of doped versus undoped Bi_2_O_2_CO_3_, it was found that N doping improved conductivity, enhanced electron transition activity, and facilitated photogenerated carrier transport (attributed to VB dispersion), thereby making Bi_2_O_2_CO_3_@Ti_3_C_2_T_x_/Polyethersulfone more effective for photocatalytic applications [110].

## 6. Other Applications of Ti_3_C_2_T_x_ MXene-Based Hybrid Photocatalysts

Beyond organic dye degradation, Ti_3_C_2_T_x_ MXene-based hybrid photocatalysts have demonstrated potential for various other applications. They have shown considerable promise in wastewater treatment, particularly in degrading organic pollutants such as dyes, pharmaceuticals, and pesticides [111]. Composite materials of Ti_3_C_2_T_x_ MXene with semiconductors such as TiO_2_ or ZnO have exhibited even greater photocatalytic efficiencies; the synergy between MXenes and these semiconductors results in improved light absorption and charge separation [112,113]. For instance, Ti_3_C_2_T_x_/TiO_2_ composites demonstrate enhanced photocatalytic activity under visible light due to the synergistic effects of both materials. These composites can efficiently degrade various dyes under sunlight, including RhB and MO, making them suitable for sustainable and cost-effective wastewater treatment [114]. Furthermore, doping MXenes with other elements or combining them with carbon-based materials like graphene can further enhance their photocatalytic properties. Nitrogen-doped Ti_3_C_2_T_x_ MXene shows improved photocatalytic degradation of antibiotics such as tetracycline under visible light, highlighting their potential in treating pharmaceutical contaminants in wastewater [115]. Ti_3_C_2_T_x_ MXene-based hybrid photocatalysts are also being explored for air purification applications, particularly in removing volatile organic compounds (VOCs) and other airborne pollutants. Ti_3_C_2_T_x_ MXene, when combined with photocatalysts like TiO_2_, can effectively degrade formaldehyde and toluene, common indoor air pollutants, under UV and visible light [116]. Incorporating noble metals like Au or Ag onto Ti_3_C_2_T_x_ MXene can further enhance their photocatalytic performance by creating localized surface plasmon resonance, increasing light absorption, and improving the degradation rates of VOCs [117]. Additionally, Z-scheme heterostructures involving Ti_3_C_2_T_x_ MXene have been developed to mimic natural photosynthesis, achieving efficient separation and transfer of photogenerated charge carriers and enhancing photocatalytic degradation of air pollutants [118]. The most applied technology is the Ti_3_C_2_T_x_ MXene-based TiO_2_ photocatalyst, which utilizes photocatalysis for glass cleaning, where UV or visible light activates TiO_2_, generating reactive oxygen species that decompose organic pollutants on the glass surface. This self-cleaning mechanism maintains transparency, reduces manual cleaning, and prevents pollutant buildup, enhancing the efficiency and durability of glass surfaces.

In addition to the degradation of dyes, the degradation of other organic pollutants, such as pharmaceutical waste and VOCs, has emerged as a challenging task requiring immediate attention [119]. In recent years, extensive studies [120] have highlighted photocatalysis as an attractive method for the efficient degradation of organic pollutants. The photocatalytic performance of a catalyst is defined by properties such as its surface-to-volume ratio, light interaction, and mechanical stability [121]. Among the variety of materials reported thus far, Ti_3_C_2_T_x_ MXenes have gained significant attention as photocatalytic materials [122]. Furthermore, the integration of Ti_3_C_2_T_x_ MXenes with other nanomaterials can significantly improve photocatalytic performance [123].

**Figure 7:**
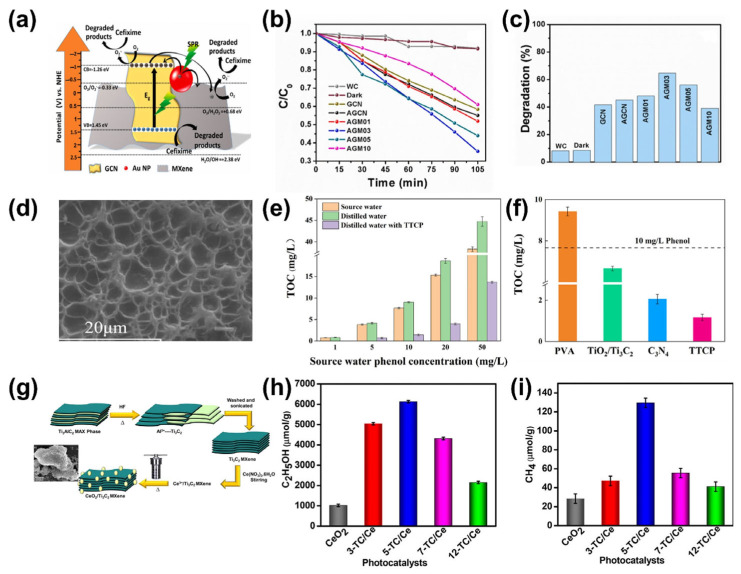
(**a**) Schematic of the cefixime degradation mechanism under light. (**b**) Kinetics of cefixime degradation. (**c**) Histogram of degradation rate (%). Reproduced with permission from [124], (**d**) SEM image of the tetracalcium phosphate (TTCP) hydrogel. (**e**) Total organic carbon (TOC) of source water under different phenol concentrations (orange column), distilled water without photocatalyst (green column), and distilled water with TTCP hydrogel (violet column). (**f**) TOC of distilled water with different catalysts. Reproduced with permission from [134]. (**g**) Schematic illustration of the synthesis process of the Ti_3_C_2_T_x_ MXene/CeO_2_ photocatalysts (inset SEM image). Comparative representation of the production of (**h**) C_2_H_5_OH and (**i**) CH_4_ under solar light illumination with different catalysts. Reproduced with permission from [135].

Kumar et al. [124] reported a novel photocatalyst composed of g-C_3_N_4_, Ti_3_C_2_T_x_, and Au nanoparticles for the degradation of cefixime. Figure 7a illustrates the degradation mechanism of cefixime decomposition under light. The composition with 3 wt% Ti_3_C_2_T_x_ demonstrated the highest degradation, achieving 64.69% cefixime removal in 105 min under visible light irradiation, as shown in Figure 7b,c. Similarly, Diao et al. [125] reported efficient photocatalytic degradation of tetracycline hydrochloride using MXene-based photocatalysts comprising g-C_3_N_4_/Ti_3_C_2_T_x_/TiO_2_. The reported ternary catalysts exhibited superior performance, chemical and photostability, and recyclability. Another MXene-based ternary photocatalyst, reported by Zhou et al. [126], facilitated photocatalytic degradation of enoxacin under visible light, where Ti_3_C_2_T_x_ improved charge separation at the interface, resulting in efficient degradation. Rdewi et al. [127] reported a ZnO-TiO_2_-MXene photocatalyst for the decomposition of carbamazepine molecules in wastewater under solar irradiation. The presented results showed 99.6% removal efficiency at pH 7, attributed to improved charge carrier transport and a reduced recombination rate due to the incorporation of TiO_2_-MXene with ZnO photocatalysts. The excellent photocatalytic performance also demonstrated reusability across multiple decomposition cycles with exceptional efficiency. Similarly, Abbas et al. [128] reported a ZnO-TiO_2_-MXene photocatalyst for the decomposition of ceftriaxone sodium molecules in water using simulated solar light.

Sukidpaneenid et al. [129] reported Ti_3_C_2_T_x_/TiO_2_ photocatalysts for the degradation of enrofloxacin antibiotics in water. The precise control over TiO_2_ loading on MXene, achieved through variations in hydrothermal processes, played a crucial role in tuning photocatalytic properties. Additionally, the intercalation of sodium ions significantly improved adsorption, and the synergistic effect of TiO_2_ loading and NaCl treatment led to the efficient removal of enrofloxacin. Shahzad et al. [130] demonstrated the degradation of carbamazepine (CBZ) under direct sunlight and UV light using Ti_3_C_2_T_x_-based heterostructure photocatalysts with {001} TiO_2_. The results showed that the Kapp value under UV irradiation was higher than under sunlight for CBZ degradation. Also, the effects of pH on degradation performance were considered. Mohanty et al. [131] reported a series of SrTiO_3_/Ti_3_C_2_T_x_-based photocatalysts decorated with Au nanoparticles for the degradation of colorless organic pollutants such as ciprofloxacin under sunlight. The significant enhancement in photocatalytic degradation for the plasmon-mediated heterostructure catalysts was attributed to the absorption of a broad solar spectrum, charge separation, and charge transport. Du et al. [132] demonstrated the photocatalytic degradation of tetracycline using CeO_2_-Ti_3_C_2_T_x_-TiO_2_ (CeMXT). The composite exhibited an excellent degradation efficiency of 94.70% for 22.19 mg/L in 104.13 min with 0.65 g/L of catalyst at pH 4.72. This excellent degradation efficiency was attributed to increased radical generation and charge separation.

Sergi et al. [133] reported a series of TiO_2_-Ti_3_C_2_T_x_ MXene photocatalysts with controlled composition for improved photocatalytic removal of benzene. The incorporation of TiO_2_ with Ti_3_C_2_T_x_ MXene enhanced optical absorption, leading to improved photocatalytic performance. The report demonstrated the potential of Ti_3_C_2_T_x_ MXenes for VOC removal and the ability of heterostructures to enhance photocatalysis. Furthermore, Huang et al. [116] reported the degradation of formaldehyde (HCHO) and acetone (CH_3_COCH_3_) using Bi_2_WO_6_/Ti_3_C_2_ (BT4) under light irradiation. Bi_2_WO_6_ photocatalysts suffer from a high recombination rate, and combination with Ti_3_C_2_ can significantly improve charge separation. Ti_3_C_2_-mediated charge separation caused charge transfer at the interface, significantly improving the photocatalytic performance of BT4. Additionally, DFT simulations indicated higher adsorption of formaldehyde and acetone on the Ti_3_C_2_ surface compared to the Bi_2_WO_6_ surface, synergistically tuning photocatalytic performance. Notably, the degradation of formaldehyde and acetone for BT4 was 2 and 6.6 times higher, respectively, than for Bi_2_WO_6_. Mo et al. [134] demonstrated photocatalytic and photothermal removal of VOCs (phenol) from water using a TiO_2_/Ti_3_C_2_T_x_/C_3_N_4_/PVA (TTCP) hydrogel under sunlight. Figure 7d shows the SEM image of TTCP. The VOC removal efficiency varied from 69.4% to 100% at different phenol concentrations (1–50 mg/L), as shown in Figure 7e,f. The membrane significantly lowered the total dissolved solids (TDSs) and TOC. The TDS level was reduced by more than two orders of magnitude, and the TOC removal efficiency was observed to be 80%.

The photocatalytic reduction of CO_2_ is another aspect of environmental remediation applications. Reducing CO_2_ into useful byproducts using light-activated catalysts represents a futuristic pathway towards sustainability [90,136]. The excellent charge separation and slow recombination rate of Ti_3_C_2_T_x_ MXene demonstrate its potential as a catalytic material for CO_2_ reduction [137]. In this regard, Cao et al. [52] demonstrated 2D/2D ultrathin Ti_3_C_2_T_x_/Bi_2_WO_6_ nanosheets prepared by in situ growth of Bi_2_WO_6_ nanosheets over Ti_3_C_2_T_x_ nanosheets. The proposed hybrid catalyst exhibited improved efficiency towards CO_2_ reduction under light due to reduced charge transfer distance, improved surface-to-volume ratio, and increased adsorption sites. The hybrid catalyst showed improved production of CH_4_ and CH_3_OH compared to Bi_2_WO_6_. Quantitatively, the production of CH_4_ increased from 0.41 μmol g^−^^1^ h^−^^1^ to 1.78 μmol g^−^^1^ h^−^^1^, while the production of CH_3_OH increased from 0.07 μmol g^−^^1^ h^−^^1^ to 0.58 μmol g^−^^1^ h^−^^1^. Similarly, Mishra et al. [135] investigated a Ti_3_C_2_T_x_-CeO_2_ hybrid catalyst with varying Ti_3_C_2_T_x_ ratios for the photocatalytic reduction of CO_2_. The schematic synthesis process for the hybrid catalysts is shown in Figure 7g. Surprisingly, the hybrid catalyst with 5 wt% Ti_3_C_2_T_x_/CeO_2_ produced 6127.04 μmol g^−^^1^ of ethanol and 129.5 μmol g^−^^1^ of methane within 5 h, significantly higher than CeO_2_, as shown in Figure 7h,i. Another report by Li et al. [137] demonstrated the potential of a Ti_3_C_2_-based hybrid catalyst comprising g-C_3_N_4_/ZnO/Ti_3_C_2_T_x_ for CO_2_ reduction into methane (CH_4_) and carbon monoxide (CO). The hybrid framework exhibited a notably higher production rate of 1.41 μmol g^−^^1^ h^−^^1^ towards CO, increased by factors of 2.7 and 1.7 compared to ZnO and g-C_3_N_4_, respectively.

Ti_3_C_2_T_x_ MXene has been extensively studied in recent years for its broad range of photocatalytic applications in environmental remediation, as evidenced by the literature. Reported results highlight the potential of Ti_3_C_2_T_x_ MXene-based catalysts for degrading dyes, pharmaceutical wastes, and VOCs, as well as for CO_2_ reduction. These photocatalysts benefit from a high surface area, efficient charge separation at the interface, and excellent light absorption, which contribute significantly to their performance.

## 7. Working Mechanism of Ti_3_C_2_T_x_ MXene-Based Hybrid Photocatalyst

The electronic structure of Ti_3_C_2_T_x_ MXene-based photocatalysts plays a crucial role in their ability to degrade targeted pollutants through photocatalysis. Generally, an ideal photocatalyst should possess a suitable bandgap, strong absorption in the visible range, prolonged charge separation lifetimes, and adequate redox potential [64]. To enhance photocatalysis efficiency, co-catalysts are employed to separate photogenerated charge carriers. When materials such as ZnO, CdS, TiO_2_, and Ti_3_C_2_ are used as co-catalysts, Schottky junctions formed with Ti_3_C_2_T_x_ facilitate the rapid dissociation of these charge carriers [138]. As discussed in the previous section, Ti_3_C_2_T_x_ MXene-based photocatalyst composites demonstrate superior photocatalytic performance compared to non-hybrid photocatalysts. This is attributed to the abundant active sites available on Ti_3_C_2_T_x_ MXene-based hybrids, their enhanced bandgap, improved light-harvesting capability, reduced charge carrier recombination, and extended photoelectron lifetime [139].

During photocatalytic degradation, Ti_3_C_2_T_x_ MXene-based hybrid photocatalysts absorb visible light, exciting photogenerated electrons into the CB and leaving holes in the VB [22]. These excited charge carriers are subsequently transferred to the Ti_3_C_2_T_x_ MXene at the interface, primarily due to the higher potential of Ti_3_C_2_T_x_. While the Schottky junction formed between n-type TiO_2_ and Ti_3_C_2_ facilitates hole transfer from TiO_2_ to Ti_3_C_2_, the inherent band bending in n-type TiO_2_ creates an energy barrier that inhibits direct electron transfer from Ti_3_C_2_ to TiO_2_ [140]. This limitation means that photogenerated electrons on Ti_3_C_2_ primarily participate in reactions with adsorbed oxygen, leading to the formation of superoxide radicals (^●^O_2_^−^). Consequently, the primary role of the Ti_3_C_2_ in this hybrid system is to act as a hole reservoir, preventing hole recombination at the Ti_3_C_2_–TiO_2_ interface, rather than directly contributing to electron transfer towards reduction reactions on the TiO_2_ surface. In this process, photogenerated electrons from the Ti_3_C_2_T_x_ MXenes migrate to the surface and react with O_2_ to produce ^●^O^2^^−^, and holes from the TiO_2_ react with hydroxyl ions (OH^−^) to produce hydroxyl radicals (^●^OH). These radicals cause the degradation of cationic and anionic dyes of organic pollutants [22,141,142].

**Figure 8:**
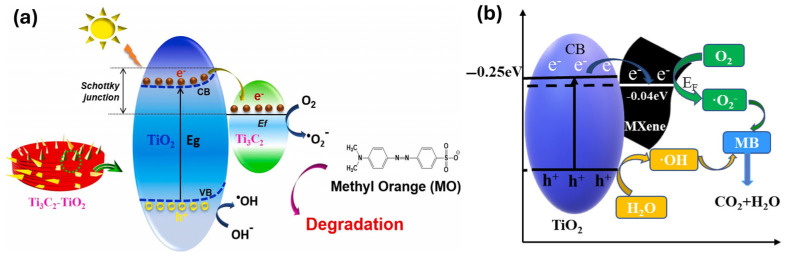
Proposed mechanisms of photocatalytic degradation: (**a**) anionic dye (MO), reproduced with permission from [114], and (**b**) cationic dye (MB) over TiO_2_/Ti_3_C_2_T_x_ composite, reproduced with permission from [72].

A photocatalytic mechanism for the Ti_3_C_2_T_x_–TiO_2_ hybrid, illustrating anionic dye (e.g., MO) degradation, is presented in Figure 8a. Initially, the light source provides high-energy photons, which activate the TiO_2_ component, generating photoinduced electrons in its CB and holes in its VB. These photoelectrons rapidly migrate from the CB of TiO_2_ to the Ti_3_C_2_T_x_ MXene, facilitated by its high electrical conductivity [73,114]. Consequently, Ti_3_C_2_T_x_ MXene accumulates a negative charge, while TiO_2_ becomes positively charged, leading to the formation of a Schottky barrier at the Ti_3_C_2_T_x_–TiO_2_ interface, which functions as a space-charge layer. Subsequently, the photogenerated electrons on Ti_3_C_2_T_x_ MXene migrate to its surface, where they react with O_2_ molecules to generate superoxide radicals (^●^O_2_^−^) [114,143]. Simultaneously, the photogenerated holes react with adsorbed OH^−^ to form hydroxyl radicals (^●^OH) [58]. These radicals play a crucial role in MO degradation. The photocatalytic reaction mechanism facilitated by the MXene-based hybrid photocatalyst (TiO_2_/Ti_3_C_2_T_x_) is illustrated in Figure 8a [114]. The mechanism can be roughly described through the following reactions:

Ti_3_C_2_–TiO_2_ + h*ϑ* → TiO_2_ (holes) + Ti_3_C_2_ (electrons) (1)

holes + OH^−^ → ^●^OH (2)

electrons + O_2_ → ^●^O^2−^ (3)

Dye (Anionic or cationic) + ^●^O^2−^ → CO_2_ + H_2_O (4)

Dye (Anionic or cationic) + ^●^OH→ CO_2_ + H_2_O (5)

For the degradation of cationic dye like MB, ^●^O^2−^ is produced through a reduction reaction between electrons and O_2_ molecules, leading to the direct degradation of MB. Additionally, light-induced holes partially oxidize MB and partially react with water to generate ^●^OH, ultimately breaking down MB [144]. As evidenced by Figure 8b, ^●^OH and holes are the primary reactive species in the TiO_2_/Ti_3_C_2_T_x_ MXene composite photocatalysis reaction system [72]. Ultimately, these radicals (^●^OH, ^●^O^2−^), possessing strong oxidizing abilities, degrade both anionic and cationic dyes, such as MO and MB molecules, directly into their oxidation products [145,146]. Therefore, a similar photocatalytic reaction mechanism occurs for both cationic and anionic dyes under light irradiation.

## 8. Challenges and Future Directions

While Ti_3_C_2_T_x_ MXene-based hybrid photocatalysts have demonstrated significant promise for organic dye pollutant degradation and environmental remediation, several challenges must be addressed to further enhance their photocatalytic performance and enable practical application.

One of the most significant challenges in MXene research is the development of green synthesis methods. Current synthesis processes often involve harsh and hazardous chemicals for etching and exfoliation, such as hydrofluoric acid, tetramethylammonium hydroxide, and tetrabutylammonium hydroxide [36]. Eliminating these chemicals is crucial for the wider applicability of MXenes.

Furthermore, the limited yield of high-quality MXene products during synthesis is another major obstacle. Current large-scale synthesis processes are inefficient, hindering commercial use. Therefore, future research efforts should focus on developing mass-production methods for high-quality, uniformly delaminated single- to few-layer MXene nanosheets. This will require a deeper understanding of the reaction kinetics and thermodynamics of the synthesis process to achieve uniform and scalable production.

The long-term stability of MXenes in harsh environmental conditions, such as oxygen, high humidity, and acidic or alkaline media, remains a significant concern [38]. Corrosion and degradation over time can compromise the catalytic efficiency of composite materials and hinder their reuse. Thermal stability is also a considerable challenge for MXene-based photocatalysts, as elevated temperatures can accelerate MXene oxidation [147].

Significant efforts have been made to address the challenges associated with MXene-based hybrid photocatalysts. The development of efficient MXene-based hybrid photocatalysts for practical applications requires a scalable synthesis of high-quality MXenes. However, achieving this remains challenging without a comprehensive understanding of the reaction kinetics and thermodynamics. Currently, the kinetics and thermodynamics of A-layer etching in MAX phases to obtain corresponding MXene layers are still underexplored [148,149,150]. Additionally, the photothermal effect of MXenes is a crucial thermodynamic factor that significantly impacts photocatalytic reactions. Upon irradiation, MXenes generate heat, raising the surrounding temperature and influencing the thermodynamics of charge carriers [151]. Accelerated thermodynamics up to an optimal temperature enhances photocatalytic efficiency; however, excessive heat can negatively impact reactant adsorption [152]. Hence, future research should focus on in-depth studies of reaction kinetics and thermodynamics to achieve uniform and large-scale MXene synthesis and MXene-based hybrid photocatalyst fabrication for practical applications.

Environmentally friendly, large-scale synthesis routes and effective antioxidation strategies for MXenes should be explored to ensure their long-term stability and sustainability. Future Ti_3_C_2_T_x_-based hybrid photocatalyst materials should also utilize their functional terminations, which serve as active sites for reaction centers, promoting covalent interactions with photocatalyst materials. This approach may enhance hybrid materials’ robustness, environmental stability, and efficiency.

To transition Ti_3_C_2_T_x_ MXene-based hybrid photocatalysts from laboratory to real-world applications, strategies for integrating them into practical systems, such as water treatment plants or portable water purification devices, should be explored. The development of user-friendly photocatalytic reactors or devices utilizing MXene-based hybrid materials for organic dye and pollutant degradation will advance these materials to the forefront of environmental remediation technologies. Conventional methods for discovering materials with desired properties are often time-consuming and complex. To address these challenges, a machine learning (ML)-driven approach can be employed to predict and understand the properties of functional materials more efficiently [153,154]. When combined with experimental data, data-driven ML models enable researchers to uncover natural correlations between catalyst properties and the degradation rates of contaminants in water resources. Significant progress has already been made in applying ML to study the catalytic activity of both standalone photocatalysts, such as ZnO [155] and TiO_2_ [156], as well as nanocomposites, including Bi_2_WO_6_/MIL-53 [155] and CuWO_4_@TiO_2_ [157], for degrading various organic dyes. However, the application of such modern artificial intelligence (AI)-based approaches in the field of MXene-based hybrid photocatalysts remains limited. To enhance the efficiency of Ti_3_C_2_T_x_ MXene-based hybrid photocatalysts, a predictive design strategy integrating AI-driven modeling with existing databases can be utilized. This methodology enables the identification and screening of optimal material combinations to enhance photocatalytic performance.

## 9. Conclusions

In summary, this review has examined the fabrication and evaluated the performance of Ti_3_C_2_T_x_ MXene-based hybrid photocatalysts for the degradation of organic dye pollutants. Ti_3_C_2_T_x_ MXene exhibits exceptional physical and chemical properties, including high electrical conductivity, excellent hydrophilicity, adsorption capability, and efficient charge transfer, while also suppressing electron–hole recombination during photocatalysis. This makes Ti_3_C_2_T_x_ MXene a promising, low-cost, and scalable alternative to noble metal catalysts, offering exceptional catalytic performance in hybrid photocatalysts. The evaluation demonstrated that Ti_3_C_2_T_x_ MXene-based hybrid photocatalysts significantly enhance dye degradation efficiency, as evidenced by increased percentage degradation and reduced degradation time, compared to non-hybrid or pure semiconducting materials. This review also provided a comprehensive understanding of the dye degradation mechanisms involving Ti_3_C_2_T_x_ MXene-based hybrid photocatalysts and highlighted various computational studies and simulations that have advanced research in this field. Furthermore, the application of ML techniques holds significant promise for optimizing the design and performance of these photocatalysts. Integrating ML into future research can accelerate the discovery of novel MXene-based photocatalysts with tailored properties. Finally, the challenges associated with Ti_3_C_2_T_x_ MXene-based hybrid photocatalysts were thoroughly identified, and future research directions were suggested to effectively address these challenges.

## Figures and Tables

**Table 1:** Selected nonhybrid photocatalysts with photocatalytic degradation performance towards cationic and anionic dyes.

Nonhybrid Photocatalysts	Dyes	Type of Dyes	Degradation Percentage (%)	References
CdS	MO	Anionic	95 (300 min)	[81]
δ-Bi_2_O_3_	MO	Anionic	98 (180 min)	[82]
TiO_2_ NPs	MO	Anionic	~95 (~120 min)	[83]
ZnO	MB	Cationic	40.88 (21 h)	[84]
TiO_2_ Hollow Nanofiber	MB	Cationic	95.2 (4 h)	[85]
CuO	MB	Cationic	62 (270 min)	[86]
Bi_2_S_2_O_3_	CR	Anionic	82 (75 min)	[87]
ZnO	CR	Anionic	97.6 (75 min)	[88]
MoSe_2_	CR	Anionic	8.44 (120 min)	[89]
g-C_3_N_4_	RhB	Cationic	75 (180 min)	[90]
BiMnO_3_	RhB	Cationic	68 (75 min)	[91]

## Abbreviations

Abbreviation	Definition
1D	One-dimensional
2D	Two-dimensional
3D	Three-dimensional
AB92	Acid blue 92
AI	Artificial intelligence
AO7	Acid Orange 7
BET	Brunauer–Emmett–Teller
CBZ	Carbamazepine
CB	Conduction band
CNTC	g-C_3_N_4_/Ti_3_C_2_
CR	Congo red
CV	Crystal violet
DI	Deionized
DMSO	Dimethyl sulfoxide
DOS	Density of state
DFT	Density functional theory
FESEM	Field emission scanning electron microscopy
g-C_3_N_4_	Graphitic carbon nitride
HF	Hydrogen fluoride
MB	Methylene blue
ML	Machine learning
PDA	Poly-dopamine
RhB	Rhodamine B
SEM	Scanning electron microscopy
TOC	Total organic carbon
TTCP	Tetra calcium phosphate
TDSs	Total dissolved solids
UV	Ultraviolet
VB	Valence band
VOCs	Volatile organic compounds
XRD	X-ray diffraction
